# Omalizumab demonstrates efficacy and safety in Chinese children under 12 with chronic urticaria: a case series

**DOI:** 10.3389/fped.2026.1815987

**Published:** 2026-04-21

**Authors:** Hui Zhao, Lei Luo, Wan-Ming Jia

**Affiliations:** 1 Department of Pediatrics, The Second Hospital of Hebei Medical University, Shijiazhuang, China; 2 Department of Pediatrics and Neonatal, Hebei General Hospital, Shijiazhuang, China; 3 Department of Cardiology, The Second Hospital of Shijiazhuang, Shijiazhuang, China

**Keywords:** chronic urticaria, efficacy, omalizumab, pediatric, safety

## Abstract

**Background:**

Omalizumab, an anti-IgE antibody, is recommended as a second-line therapy for chronic spontaneous urticaria in patients aged ≥12 years; however, real-world evidence for its use in younger Chinese children remains limited. Therefore, this study aimed to evaluate its clinical efficacy, safety, and associated characteristics of omalizumab in this underserved pediatric population under 12 years of age.

**Methods:**

We conducted a retrospective analysis of 13 Chinese children (aged <12 years) with refractory chronic urticaria treated with omalizumab. Effectiveness was assessed by the Urticaria Activity Score over 7 days (UAS7), and safety was evaluated by monitoring adverse events.

**Results:**

Of the 13 pediatric patients (median age 5.8 years; 46.2% male), all demonstrated rapid clinical improvement within one week after the initial omalizumab dose. Oral antihistamines were successfully discontinued in all patients within four weeks, accompanied by a significant reduction in the median UAS7 score from 20.0 at baseline to 4.0 (*p* = 0.001). All 74 administered doses were well tolerated, with no adverse events reported; notably, nearly half of the cohort had comorbid allergic conditions.

**Conclusion:**

The rapid symptom control and excellent safety profile observed support omalizumab as an effective and well-tolerated therapeutic option for children under 12 with chronic urticaria.

## 1 Introduction

Chronic urticaria (CU) is defined as the occurrence of wheals, angioedema, or both for more than 6 weeks. Chronic inducible urticaria is induced by specific triggers. When the triggers are not definite, it is named chronic spontaneous urticaria (CSU) (1). The point prevalence of CU is 1.43% in children (age 0–19), and there is no sex-specific difference in children less than 15 years (2). The resolution rate in children with CU is low (10.3 per 100 patient-years), and the most common type is CSU (78.0%) (3).

According to current guidelines, the first-line treatment for children with CU is 2nd generation H1-antihistamines. If patients fail to respond to high doses of antihistamines, the anti-IgE antibody omalizumab is recommended as a second-line therapeutic option for adults and adolescents with CSU who are 12 years and older (1). And clinical studies have demonstrated that omalizumab is efficacious against most types of chronic inducible urticaria (4). However, there are limited data available about the efficacy and safety of omalizumab treatment in children with CU less than 12 years. Therefore, the aim of this paper was to describe our experience of using omalizumab in pediatric patients under 12 years with CU.

## 2 Methods

### 2.1 Patients and study design

This retrospective study analyzed clinical data from pediatric patients with CU who were treated with omalizumab at the Pediatric Allergy and Immunology Clinic of the Second Hospital of Hebei Medical University between January 2022 and December 2024. The study protocol was approved by the Institutional Review Board of the Second Hospital of Hebei Medical University (Approval No. 2025-R678).

Patients were enrolled in the study if they met all the following criteria: (1) aged <12 years with a confirmed diagnosis of CU; (2) inadequate response to high-dose oral antihistamines (defined as at least twice the standard licensed dose); (3) having received at least two doses of omalizumab treatment. And patients were excluded from the study based on the following criteria: (1) age ≥ 12 years; (2) presence of comorbid autoimmune disorders or other clinically significant systemic diseases; or (3) concurrent use of immunosuppressive agents or other biologic therapies.

Following obtainment of written informed consent from all legal guardians, a 150 mg dose of omalizumab was administered via subcutaneous injection. Baseline characteristic, including demographic information, allergy comorbidities, Urticaria Activity Score over 7 days (UAS7), and total immunoglobulin E (IgE) levels, along with treatment outcomes (dates of omalizumab administration, the week-4 UAS7 score and adverse events) were retrospectively collected from the electronic medical record system. Well-controlled disease was defined as a UAS7 score ≤6 at week 4, a widely accepted threshold for disease control in chronic urticaria (1).

### 2.2 Statistical analysis

Statistical analysis was performed using IBM SPSS Statistics (version 26.0, IBM Corp., Armonk, NY, USA, RRID:SCR_016479). Normality was assessed using the Shapiro–Wilk test. Due to non-normal distribution, continuous data were presented as median and interquartile range (IQR, 25th– 75th percentile). Differences in UAS7 scores before and after treatment were analyzed using the Wilcoxon signed-rank test. A *p*-value <0.05 was considered statistically significant.

## 3 Results

A total of 13 pediatric patients receiving omalizumab treatment were included in this study. The cohort consisted of 6 males (46.2%). The median age at presentation was 5.8 years (IQR: 5.5–7.1), while the median age at symptom onset was 5.3 years (IQR: 3.0–5.8). The median duration of CU prior to the initiation of omalizumab therapy was 12.0 months (IQR: 3.5–36.0). One patient presented with angioedema, and nearly half of the patients had comorbid allergic conditions, including atopic dermatitis, cow's milk protein allergy, allergic rhinitis, or asthma. The baseline characteristics of the patients are summarized in Table 1.

**Table 1:** Baseline characteristics of the study cohort.

Variable	*N* = 13
Male *n* (%)	6 (46.2)
Age (years)^a^	5.8 (5.5–7.1)
Age at symptom onset (years)^a^	5.3 (3.0–5.8)
Symptom duration at admission(months)^a^	12.0 (3.5–36.0)
Concomitant angioedema, *n* (%)	1 (8.0)
Concomitant allergic disease, *n* (%)	7 (53.8)
Atopic dermatitis	1 (8.0)
Cow milk protein allergy	1 (8.0)
Allergic rhinitis	4 (30.8)
Asthma	1 (8.0)
Total IgE (IU/mL)^a^	59.4 (30.4–277.1)

IQR, interquartile range.

a Median.

Four patients experienced disease exacerbation following infection, while no clear predisposing factors were identified in the remaining cases. Five patients received supplemental corticosteroids (oral or intramuscular) concurrently with oral antihistamines prior to omalizumab initiation. All 13 pediatric patients showed significant symptom improvement within the first week after the initial injection and completely discontinued oral medications within 4 weeks. By week 4, the median UAS7 score decreased significantly from a baseline of 20.0 (IQR: 18.0–26.5) to 4.0 (IQR: 2.5–5.5; *p* = 0.001). At week 4, 11 of the 13 patients (84.6%) achieved well-controlled disease (UAS7 ≤ 6), while the remaining two patients had a UAS7 of 7, indicating mild residual disease activity. A total of 74 subcutaneous injections were administered, with no adverse events reported. Individual patient responses to omalizumab therapy are presented in Table 2.

**Table 2:** Omalizumab response by patient.

Patient	Gender	Age (y)	Symptom dur., mo	Total IgE (IU/mL)	No. of injections	UAS7_Wk0_	UAS7_Wk4_
1	F	6.8	12	48.7	3	21	4
2	M	10.3	60	245.3	11	28	6
3	M	5.5	24	29.3	3	20	3
4	M	3.6	12	77.0	2	18	4
5	M	5.8	3	59.4	6	18	5
6	F	2.9	24	7.1	3	16	1
7	M	8.3	60	324.0	14	30	7
8	F	7.1	2	31.4	8	20	5
9	F	5.5	48	25.3	3	22	2
10	M	5.8	2	1,000.0	3	19	4
11	F	7.1	6	127.6	5	25	3
12	F	5.8	4	309.0	6	32	7
13	F	5.8	24	46.0	7	16	1

## 4 Discussion

In our case series, 11 patients (84.6%) achieved well-controlled disease (UAS7 ≤ 6) by week 4, while the remaining two patients had a UAS7 of 7, indicating mild residual disease activity but not complete control. Of note, both had a high baseline UAS7 (>30) and comorbid allergic rhinitis. Prior to omalizumab, all patients had moderate-to-severe CU despite double-dose antihistamines (baseline UAS7: 16–32); median disease duration was 12.0 months (IQR: 3.5–36.0). Only one had a history of angioedema, precluding meaningful subgroup analysis. Additionally, five patients (38.5%) had required systemic corticosteroids prior to enrollment, consistent with previous studies showing that omalizumab is an effective corticosteroid-sparing treatment (5).

Interestingly, patients with baseline UAS7 16–30 all achieved well-controlled disease, whereas the two with baseline >30 achieved only a partial response, suggesting that greater baseline severity may be associated with a less complete response within the initial 4 weeks. Nevertheless, a UAS7 of 7 represents a substantial improvement from baseline >30, indicating meaningful clinical benefit. This finding aligns with studies showing that patients with higher baseline UAS7 are less likely to achieve complete control and may require longer treatment or dose optimization (6, 7). Notably, both patients with partial response at week 4 subsequently achieved well-controlled disease with continued treatment, highlighting that initial incomplete response does not preclude eventual success. Overall, all patients derived meaningful clinical benefit from omalizumab, with most achieving well-controlled disease within four weeks, supporting its use even in patients with moderate-to-severe disease. No differences in response were observed based on prior corticosteroid use or disease duration, likely due to the small sample size.

In addition to its efficacy, omalizumab exhibited an excellent safety profile in this pediatric case series (aged <12 years). That is consistent with the results reported by Ari (8) and Al-Shaikhly (9). Furthermore, omalizumab has demonstrated a safe profile in children aged 6–<12 years with moderate-to-severe allergic asthma (10). In support of this, a recent meta-analysis demonstrated a favorable safety profile for omalizumab, showing no significant increase in adverse events compared to placebo among asthmatic children aged 6–11 years (11). These findings suggest that omalizumab can be an effective and safe treatment option for CU in this pediatric population.

Notably, our study included eight children under the age of 6 years, with the youngest participant aged 2.9 years, providing valuable safety and efficacy data in this underrepresented population. This youngest patient (Patient #6 in Table 2) successfully received 3 omalizumab injections at 6-week intervals without any adverse events. Omalizumab has been shown to be safe and effective in children as young as 1 year of age with food allergy, as demonstrated in the pivotal OUtMATCH study (12). However, its efficacy and safety in children under 2 years of age with chronic urticaria remain largely unexplored, highlighting the need for further studies to establish optimal dosing and safety in this younger pediatric population.

In our case series of 13 children with CU, 7 (53.8%) had allergic diseases. A large population-based study by James et al. (13) revealed high comorbidity rates in CU patients, with allergic rhinitis (47.9%), asthma (21.2%), and other allergies. However, another study reported a significantly lower prevalence of asthma and allergic rhinitis in CU patients compared to the general population (*P* < 0.001) (14). Similar contradictory findings have been reported in studies on pediatric CU. A study of children with CSU revealed a higher prevalence of atopic diseases compared to the general pediatric population, suggesting a possible TH2-associated urticaria subtype (15). In contrast, a study from South Korea reported no significant differences in the prevalence of allergic diseases or total IgE levels between the CU and control groups (16).

Prior to omalizumab treatment, Patients #2 and #7 (Table 2) each had a 5-year history of chronic urticaria accompanied by allergic rhinitis. They received 11 and 14 injections respectively, with the dosing interval gradually extended to 8 weeks based on the absence of urticaria recurrence during stepwise prolongation (e.g., from 4 to 5 weeks, to 6 weeks, and finally to 8 weeks). Concurrently, the patient's concomitant allergic rhinitis was also well-controlled throughout the omalizumab treatment period. The two cases indicate that an extended dosing interval may also be feasible in pediatric patients, aligning with existing studies conducted in adults (17). Although limited by the retrospective design and small sample size, they provide valuable preliminary evidence for refining omalizumab treatment strategies. Prospective large-scale studies are warranted to confirm these findings and establish personalized, cost-effective long-term management strategies.

Higher based IgE levels may predict better response to omalizumab. A study by Ertas et al. showed that CSU patients with baseline total IgE <43 IU/mL had poorer responses to omalizumab (33% non-response at 12 weeks), whereas only 5% of those with IgE >43 IU/mL failed to respond (18). And, Straesser et al. (19) found that patients who did not respond to omalizumab had significantly lower IgE levels compared to partial and complete responders. As total serum IgE levels can be affected by multiple factors including age, sex, race, season and assay methods etc, standardized reference ranges for pediatric age groups remain unavailable. All patients in this case series showed excellent responses to omalizumab regardless of their total IgE levels (ranging from 7.1 IU/mL to 1,000.0 IU/mL). The predictive value of total IgE levels for omalizumab treatment response in pediatric CU patients aged <12 years requires further investigation.

An interesting observation in our study was the rapid clinical improvement within one week of the initial omalizumab dose, which was observed in all 13 patients, regardless of their baseline total IgE levels. This rapid onset cannot be fully explained by the classical mechanism of Fc*ε*RI downregulation, which typically requires weeks (20). Rather, it likely involves the immediate neutralization of free IgE function (21). This rapid depletion of free IgE may deprive pathogenic IgG autoantibodies, which are common in the autoimmune endotype of CSU, of their target (22, 23), thereby contributing to the immediate stabilization of mast cells and basophils. This mechanism aligns with the “scavenger hypothesis” proposed for omalizumab's action in autoimmune urticaria (21).

Infection is a common cause of urticaria in children. Patients #4 and #9 (Table 2) both experienced exacerbation of urticaria following infection and omalizumab was initiated after resolution of the acute episode and administered at 12-week intervals; this longer dosing interval was selected based on the timing of rash recurrence (approximately 12 weeks in these two patients), thereby providing sustained disease control while reducing dosing frequency. This extended dosing approach is consistent with previous reports suggesting that some patients may tolerate longer intervals (24). Data on the use of omalizumab in cases of infection-triggered urticaria exacerbation are still quite limited. The two cases presented here demonstrate that omalizumab, when initiated after resolution of acute infection and followed by subsequent dosing at extended intervals, can lead to significant clinical improvement. These experiences contribute meaningful practical insight and suggest a potential role for longer-interval omalizumab dosing in similar pediatric cases. Further studies are warranted to confirm its efficacy and optimize treatment protocols.

In summary, our findings demonstrate that omalizumab is an effective and well-tolerated therapy for CU in children aged <12 years. Nevertheless, further validation through large-scale randomized controlled trials remains necessary.

## Data Availability

The original contributions presented in the study are included in the article/Supplementary Material, further inquiries can be directed to the corresponding authors.
